# Comparison between Glasgow prognostic criteria and O-POSSUM/ P-POSSUM physiological indices in patients undergoing gastrectomy for gastric adenocarcinoma and the occurrency of early postoperative complications

**DOI:** 10.1590/0100-6991e-20243662-en

**Published:** 2024-06-14

**Authors:** WILLIAM FREDERIC DE ARAÚJO WILLMER, EDGAR FREITA NDUNDUMA SAMONGE, OSWALDO ESTEVES BARCIA, GUSTAVO MAGALHÃES BOGOSSIAN, LIA ROQUE ASSUMPÇÃO, RUY GARCIA MARQUES

**Affiliations:** 1 - Hospital Universitário Pedro Ernesto/UERJ, Programa de Pós-graduação em Fisiopatologia e Ciências Cirúrgicas - Rio de Janeiro - RJ - Brasil; 2 - Instituto de Pós-graduação Médica Carlos Chagas (IPGMCC) - Rio de Janeiro - RJ - Brasil; 3 - Universidade Estácio de Sá (UNESA) - Rio de Janeiro - RJ - Brasil; 4 - Universidade do Grande Rio (Unigranrio) - Rio de Janeiro - RJ - Brasil

**Keywords:** Inflammation, Gastrectomy, Prognosis, Stomach Neoplasms, Neoplasias Gástricas, Inflamação, Gastrectomia, Prognóstico

## Abstract

**Introduction:**

Gastric cancer is still the third cause of death worldwide due to malignant neoplasms. Its prognostic indices have not yet been well defined for surgical intervention in terms of stratifying the intensity of chronic inflammation. The Glasgow Prognostic Score (GPS) and O-POSSUM and P-POSSUM Indices may constitute these standardizations and were tested to assess the association between them and the prognosis after curative gastrectomy.

**Method:**

Retrospective observational study, analysing medical records of patients with gastric adenocarcinoma who underwent gastrectomy, from 2015 to 2021, in two hospitals in Rio de Janeiro. Surgical extension, pre, peri and postoperative clinical and laboratory data were observed, up to 30 days after surgery. Patients were layered by GPS and compared according to the Clavien-Dindo (CD) classification. Logistic regression was performed to test the association between the outcome and independent variables.

**Results:**

Of the 48 patients, 56.25% were female. There was difference between the groups regarding surgical extension and GPS (both with p<0.001), while O-POSSUM, P-POSSUM and age showed no difference. Factors associated with CD ≥ III-a complication in the univariate analysis were GPS (OR: 85,261; CI: 24,909- 291,831) and P-POSSUM (OR: 1,211; CI:1,044-1,404). In the multivariate analysis, the independent factors associated with CD ≥ III-a were GPS (OR:114,865; CI: 15,430-855,086), P-POSSUM (OR: 1,133; CI: 1,086-1,181) and O-POSSUM (OR: 2,238; CI: 1,790-2,797).

**Conclusion:**

In this model, GPS, P-POSSUM and O-POSSUM predicted serious surgical complications. There is a need for further studies to establish strategies to minimize the inflammatory response in the preoperative period.

## RESUMO

**Introdução:**

O câncer gástrico segue como terceira causa de mortalidade mundial por neoplasias malignas. Seus índices prognósticos ainda não foram bem definidos para intervenção cirúrgica quanto à estratificação da intensidade da inflamação crônica. Os Critérios Prognósticos de Glasgow (CPG) e os índices de O-POSSUM e PPOSSUM podem constituir essas padronizações e foram testados para avaliar a associação entre eles e o prognóstico após gastrectomia curativa.

**Método:**

Estudo retrospectivo, analisando prontuários de pacientes com adenocarcinoma gástrico e submetidos à gastrectomia, no período de 2015 até 2021, em dois hospitais no Rio de Janeiro. Foram observados a extensão cirúrgica, os dados clínicos e laboratoriais pré, peri e pós-operatórios, até 30 dias após a cirurgia. Os pacientes foram estratificados pelos CPG e comparados segundo classificação de ClavienDindo (CD). Regressão logística foi realizada para testar associação entre o desfecho e variáveis independentes.

**Resultados:**

Dos 48 doentes, 56,25% eram do sexo feminino. Houve diferença entre os grupos quanto à extensão cirúrgica e CPG (ambos com p<0,001), enquanto O-POSSUM, P-POSSUM e idade não apresentaram diferença. Fatores associados com complicação CD ≥ III-a na análise univariada foram CPG (OR: 85,261; IC: 24,909-291,831) e P-POSSUM (OR: 1,211; IC: 1,044-1,404). Na análise multivariada, os fatores independentes associados ao CD ≥ III-a foram CPG (OR: 114,865; IC: 15,430-855,086), P-POSSUM (OR: 1,133; IC: 1,086-1,181) e O-POSSUM (OR: 2,238; IC: 1,790-2,797).

**Conclusão:**

Neste modelo, CPG, P-POSSUM e O-POSSUM previram complicações cirúrgicas graves. Há necessidade de estudos mais aprofundados para instituir estratégias de forma a minimizar a resposta inflamatória no período pré-operatório.

## INTRODUCTION

Gastric cancer is the fifth most common type of cancer in the world and the third cause of mortality in both sexes. The peak incidence occurs in men, around 60 years of age^1^. The main risk factor is chronic inflammation associated with Helicobacter pylori infection, and high sodium and alcohol consumption, smoking, and age also play a role^2^ ^-^ ^7^.

Early diagnosis is limited, as most patients display advanced-stage symptoms at the time of presentation^2^ ^-^ ^4^. Surgical treatment with curative intent consists of total or subtotal gastrectomy and eventual resection of adjacent organs and extensive lymphadenectomies. These procedures can influence possible postoperative complications, thus constituting additional poor prognosis factors for gastric cancer^8^ ^,^ ^9^.

Identification and stratification of the intensity of the inflammatory response in these patients can help in customizing therapy (surgical or not), since the exacerbated inflammatory response is associated with high rates of surgical complications. The treatment of such complications and their resulting hospitalizations are very costly for the health system^10^ ^,^ ^11^.

The Glasgow Prognostic Criteria (GPC) were initially defined to evaluate the intensity of the inflammatory response in patients diagnosed with malignant neoplasia, mainly in cases arising from the digestive tract. This assessment is carried out by attributing values to changes in C-reactive protein (CRP) and albumin^12^.

Patients with an assessment of 0 (CRP <10mg/l and albumin >3.5g/dl) have a postoperative morbidity and mortality rate of around 10%; with a score of 1 (CRP >10mg/l or albumin <3.5g/dl), morbidity and mortality is around 30% to 40%; and patients scoring 2 (CRP >10mg/l and albumin <3.5g/l) have a risk of postoperative complications an death greater than 60%. Recently, these criteria were incremented with the assessment of the neutrophil/lymphocyte, platelet/lymphocyte, and monocyte/lymphocyte ratios, with the aim of improving the characterization of the intensity of the inflammatory response^11^.

The Physiological and Operative Severity Score for the enUmeration of Mortality and morbidity (POSSUM) was proposed as a way of standardizing data on patients undergoing surgical treatment. The score considers both the physiological (P-POSSUM) aspects of the patient at admission, with 12 variables, and the severity of the operation (O-POSSUM) performed, with six other variables^13^.

The objective of this study was to test the association between the Glasgow criteria, O-POSSUM and P-POSSUM, to verify the outcome of Clavien-Dindo complications greater than III-a in patients undergoing gastrectomy for gastric adenocarcinoma with curative intent.

## METHODS

This is a retrospective, observational study, carried out from 01/01/2015 to 12/31/2021, at the Hospital Federal da Lagoa and the Hospital Universitário Pedro Ernesto, both located in the city of Rio de Janeiro. The study was approved by the Plataforma Brasil Ethics Committee, under opinion number 5.782.089 and CAAE: 6488 1422.3.0000.5259.

Initially, we selected 160 medical records of patients diagnosed with gastric adenocarcinoma who underwent gastrectomy with curative intent, 42 from Hospital Federal da Lagoa and 118 from Hospital Universitário Pedro Ernesto.

We included all patients with gastric cancer proven by upper endoscopy and with a histopathological diagnosis of adenocarcinoma, whose charts had records of all physiological and surgical criteria necessary to perform the P-POSSUM/O-POSSUM analysis in the perioperative period, who had undergone laparoscopic or laparotomic total or subtotal gastrectomy with coloepiploic detachment plus lymphadenectomy of the perigastric lymph nodes up to gastroepiploic ligation and omentectomy. Duodenal section was performed with a stapler and reinforcement with Prolene 3.0 suture, as well as lymphadenectomy of the omental bursa and gastrohepatic ligament, with ligation of the right gastric and gastroduodenal arteries at their origin, periaortic and celiac trunk lymphadenectomy, and left gastric ligation. In the subtotal gastrectomies, gastric section was performed, and in the total ones, cardia lymphadenectomy and ligation of the short vessels. Roux-en-y reconstruction was performed with Stapler + Prolene 3.0, and patients were followed up until at least the first 30 postoperative days, through their medical records.

**Figure 1:**
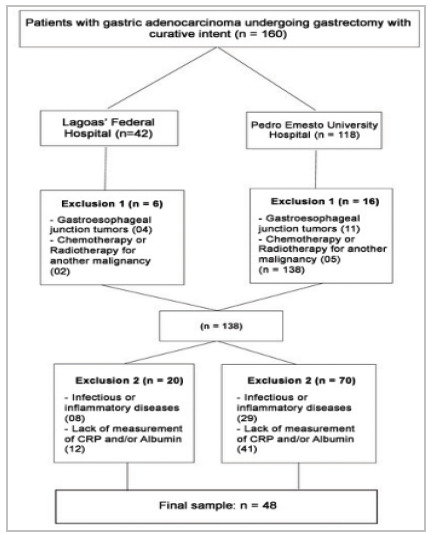
Casuistry and application of inclusion and exclusion criteria.

After applying the inclusion and exclusion criteria, 48 patients were eligible for this study (Figure 1).

### Criteria analyzed

The Glasgow Prognostic Criteria data were collected in the preoperative period, the last examination carried out before the surgical procedure, while the P-POSSUM and O-POSSUM scores were based on examinations carried out immediately after the procedure. We correlated these data with outcomes occurring within 30 days after the surgical procedure.

We used the surgical risk stratification of the American Society of Anesthesiologists (ASA), which does not consider the procedures to be performed and refers to the presence of previous clinical comorbidities, whether controlled or not. In this study, we calculated this classification in parallel. While the P-POSSUM (physiological) scoring index evaluates laboratory parameters, the O-POSSUM (oeprative) evaluates intraoperative parameters. Scores from both POSSUM criteria are entered into two complex formulas with mathematical calculations, which can predict morbidity and mortality risks. These criteria result in a direct measurement in absolute numbers, adding up the score

### Groups according to morbidity and mortality

- CD < III-a (complications without the need for intervention)
  - CD grade I: Any deviation from the ideal postoperative course without the need for pharmacological treatment or surgical, endoscopic, or radiological interventions. The permitted therapeutic regimens are antiemetic drugs, antipyretics, analgesics, diuretics, electrolytes, and physiotherapy. This category also includes surgical wounds drained at the bedside;
  - CD grade II: Complication that requires pharmacological treatment with drugs other than those permitted for grade I complications. Blood transfusion and total parenteral nutrition are also included;
- CD ≥ III-a (complications requiring intervention)
  - CD grade III: Complication requiring surgical, endoscopic or radiological intervention;
  - CD grade III-a: Intervention without general anesthesia;
  - CD grade III-b: Intervention under general anesthesia;
  - CD grade IV: Life-threatening complication, including of the Central Nervous System*. There is need for ICU admission. * Brain hemorrhage, ischemic stroke, subarachnoid bleeding, excluding transient ischemic attacks;
  - CD grade IV-a: Single organ dysfunction (including dialysis);
  - CD grade IV-b: Multiple organ dysfunction;
  - CD grade V: Death.

This stratification made it possible to use binomial logistic regression, facilitating the interpretation of statistical data.

### Statistical analysis

In statistical analysis, we expressed continuous variables as mean ± standard deviation (SD) or median and interquartile range, depending on whether they were normally distributed or not. Categorical variables were expressed as percentages.

We used the T test for normally distributed variables, the Mann-Whitney non-parametric sum ranking test for variables without normal distribution, and the chi-square test to compare percentages.

We performed a logistic regression grouped by hospital to test the association between the binary outcome (having or not having CD ≥ III-a) and independent variables, such as the Glasgow, O-POSSUM and P-POSSUM scores, in addition to total versus subtotal gastrectomy.

We used an alpha error of 0.05 to determine statistical significance and study power of 80% (beta error).

We utilized the Stata software version 17.0 (StataCorp LP, College Station, TX) for statistical analysis.

## RESULTS

Table 2 contains the clinical-demographic characteristics and their differences regarding the analyzed outcome. Of the 48 patients included in the study, the majority were female, with 27 women (56.25%). Age ranged from 60 to 70 years, with an average of 63.72 years. The average age of individuals who presented with CD ≥ III-a complications was 69.27 years. Of the female patients, 12.50% had CD ≥ III-a complications, and among males, 10.41%. Regarding surgical extension, total gastrectomies (TG) represented 45.83% (22) of cases, of which 12.5% (six) had CD ≥ III-a complications. There was a difference in the CD ≥ III-a complication group, with a higher proportion for TG and ASA III, (p<0.001 and p=0.074, respectively), as well as P-POSSUM and TG (p<0.001 and p=0.065, respectively).

[Table t1]

**Table 1 t1:** P-POSSUM / O-POSSUM score ^13^

Critérios de O-POSSUM/P-POSSUM^13^	
Physiological variables	Surgical variables
Age	Operative severity
Cardiac signs	Multiple procedures
Respiratory signs	Blood loss
Electrocardiography	Peritoneal contamination
Systolic blood pressure	Malignant dissemination status
Heart rate	Mode of surgery (elective/urgent)
Hemoglobin	
Leukocytes	
Urea	
Sodium	
Potassium	
Glasgow Coma Scale	

**Table 2:** Clinical demographic data grouped by Clavien-Dindo complications.

Variáveis	CD ≥ III-a	CD < III-a	Total	p-value
ASA	I 0 (0%)	I 5 (10,81%)	48	0,074
	II 35 (72,73%)	II 40 (83,78%)		
	III 13 (27,27%)	III 3 (5,41%)		
Age (mean)	69,27	62,54	63,72	0,902
Sex	M 5 (10,41%)	M 16 (33,34%)	48 (100%)	
	F 6 (12,5%)	F 21 (43,75%)		
TG	6 (12,50%)	16 (33,33%)	22 (45,83%)	< 0,001
P-POSSUM	37 (77,08%)	11 (22,92%)	48	0,065
O-POSSUM	37 (77,08%)	11 (22,92%)	48	0,938
GPC 0	0 (0%)	18 (100%)	18	< 0,001
GPC 1	2 (10%)	18 (90%)	20	
GPC 2	9 (90%)	1 (10%)	10	

ASA: American Society of Anesthesiologists surgical risk stratification. TG: Total Gastrectomy. GPC: Glasgow Prognostic Criteria. CD: Clavien-Dindo.

**Figure 2:**
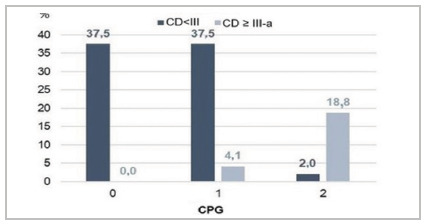
Relationship between GPC and postoperative complications.

The stratification of patients according to the Glasgow Prognostic Criteria resulted in 37.50% (18 patients) with GPC 0 presenting CD < III-a, 41.6% (20) with GPC 1, and 20.8% (10) with GPC 2. Of the last two subgroups, 4.1% (two) and 18.8% (nine), respectively, had surgical complications requiring intervention or admission to an intensive care unit (CD ≥ III-a). There were no deaths recorded in the first 30 days after surgery (Figure 2).

[Fig f3]

**Figure 3 f3:**
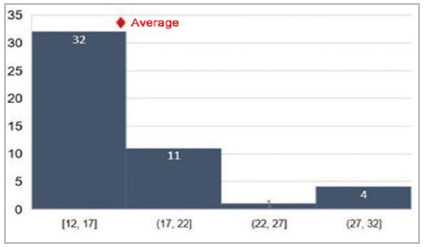
Average morbidity observed by P-POSSUM.

[Fig f4]

**Figure 4 f4:**
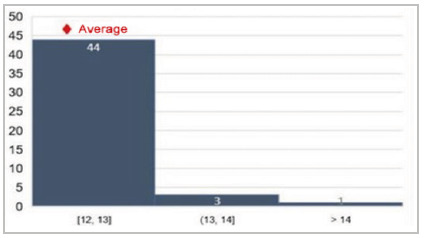
Average morbidity observed by O-POSSUM.

[Fig f5]

**Figure 5 f5:**
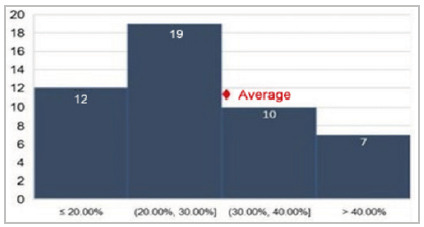
Average morbidity of the POSSUM Physiological Index.

[Fig f6]

**Figure 6 f6:**
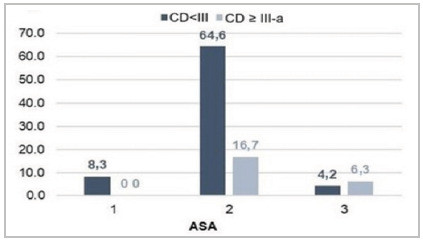
Relationship between postoperative complications and ASA classification.

In addition to age, we also calculated the means of GPC (0.83), P-POSSUM (16.95), and O-POSSUM (2.25), obtaining an estimate of morbidity using the POSSUM parameter, on average of 30.82%.

As for risk stratification with the ASA classification, all ASA I patients (8.3%) had CD < III-a complications; in the ASA II and III groups, 16.7% and 6.3% had CD ≥ III-a complications versus 64.6% and 4.2% with CD < III-a complications, respectively. ASA IV or V scores were not present, since these contraindicate the surgical procedure.

When using univariate regression, the variables P-POSSUM (OR 1.211, 95% CI 1.044-1.404) and GPC (OR 85.261, 95% CI 24.909-291.831) were able to separately predict CD ≥ III-a complications, while O-POSSUM (OR 0.897, 95% CI 0.398-2.021) and TG (OR 1.575, 95% CI 0.407-6.0 95) were not associated with the outcome when tested in isolation (Table 3).

**Table 3:** Univariate analysis of prognostic scores and performance of total gastrectomy, in relation to the outcome CD ≥ III-a.

Variables	OR	95% CI	p-value
GPC	85,261	24,909-291,831	0,001
P-POSSUM	1,211	1,044-1,404	0,011
O-POSSUM	0,897	0,398-2,021	0,792
TG	1,575	0,407-6,095	0,511

GPC: Glasgow Prognostic Criteria. TG: Total Gastrectomy. CI: Confidence Interval.

In the multivariate analysis, there was an independent association of the complication outcome CD ≥ III-a with the variables GPC (OR 114.865, 95% CI 15.430-855.086), O-POSSUM (OR 2.238, 95% CI 1.790-2.797), and P-POSSUM (OR 1.133, 95% CI 1.086-1.181), as shown in Table 4. Despite presenting proportionally more complications, total gastrectomy showed no significant association.

**Table 4:** Multivariate analysis of prognostic scores and Total Gastrectomy in relation to the outcome CD ≥ III-a.

Variables	OR	95%CI	p-value
GPC	114,8651	15,430-855,086	<0,001
P-POSSUM	1,133	1,086-1,181	<0,001
O-POSSUM	2,238	1,790-2,797	<0,001
TG	0,888	0,134-5,898	0,902

GPC: Glasgow Prognostic Criteria. TG: Total Gastrectomy. CI: Confidence Interval.

## DISCUSSION

The study showed a relationship between GPC positivity and the outcome of complications (CD ≥ III-a). With each point increase in the score, the chance of CD ≥ III-a complications increases 85.281 times as per the univariate analysis and 114.86 times by the multivariate model. Kubota et al. evaluated the systemic inflammatory response with GPC and the severity of postoperative complications with the Clavien-Dindo classification in 1,017 patients after curative resection of gastric cancer. The authors showed that GPC was not associated with the incidence of complications (p=0.9289) and that 163 patients (16.0%) had postoperative complications of CD ≥ II14. The non-convergence with our results may occur due to the specificity of the relationship between GPC and complications that require interventions (CD ≥ III-a), a milestone that is of interest to the surgeon and that may also constitute an additional prognostic factor for cancer gastric after curative resection. This strong association between GPC and incidence of complications (CD ≥ III-a) can efficiently, quickly, and simply predict the probability of the appearance of serious surgical complications, without complex mathematical calculations. Therefore, this association can be used in prognostic assessment in surgical procedures with curative intent^15^ ^,^ ^16^.

Our data indicate that GPC and P-POSSUM were associated with the outcome CD ≥ III-a in univariate analysis. When analyzed in a multivariate model, GPC and the POSSUM indices (P-POSSUM and O-POSSUM) were independent predictors of CD ≥ III-a. The extent of the surgical procedure (total vs. subtotal gastrectomy) was not associated with the outcome CD ≥ III-a in the univariate analysis, and in the multivariate analysis TG was not an independent predictor of CD ≥ III-a. Regarding the access used in the surgical procedure (laparoscopic vs. open gastrectomy), we cannot relate this variable with a CD ≥ III-a complication, despite the laparoscopic access lower rate of postoperative complications, shorter hospital stay, and quicker recovery. Laparoscopic access is reserved for early cases, lower patient morbidity, and less complicated patient selection^17^ ^-^ ^19^. In contrast, robotic gastrectomy, when compared with laparoscopic gastrectomy for gastric cancer, presents advantages, both operative (operative time, estimated blood loss, number of lymph nodes recovered) and perioperative (time to first flatulence, time to restart oral intake, length of stay, Clavien-Dindo (CD) ≥ III complications, pancreatic complications), in the absence of clear differences in oncological results^20^ ^-^ ^22^.

Age did not show significance, as in five other cohort studies mentioned in the systematic review and meta-analysis carried out by Figueiredo et al., in which 255 publications were identified and 15 studies analyzed^15^. However, in the retrospective review of 650 patients undergoing elective surgery for gastric cancer conducted by Ishizuka et al., GPC was associated with age (≤70/>70 years), with an odds ratio of 2.348 (95% CI 1.570-3.511)^23^.

In general, the literature shows that the O-POSSUM/P-POSSUM indices overestimate the risk of morbidity and mortality, generating estimates of the appearance of complications well above those presented. An example can be seen in the five-year retrospective review of the cases of 81 patients with gastric adenocarcinoma who underwent surgery, in which P-POSSUM predicted double (12.4%) the observed mortality (6.2%) and overestimated post-operative complications, especially in higher risk groups, with an observed incidence of 33.3% versus the expected 63%^9^. This was also observed by Carvalho-e-Carvalho et al. in patients undergoing surgical procedures for colorectal cancer, their actual morbidity being 15.6%, as opposed to the expected morbidity of 39.2% according to P-POSSUM. Our analysis confirmed these data and our sample follows a similar distribution pattern, despite there being no records of deaths within 30 days after surgery^14^ ^,^ ^24^ ^-^ ^27^.

With the results of this study, we can suggest the use of possibilities to reduce patients’ inflammatory state in the preoperative period, for example using immunonutrition, although its possible benefits in reducing complications after major surgical procedures for gastrointestinal cancer in general has not been reproduced in patients undergoing gastrectomy^17^. Thus, although one cannot exclude a benefit, there is currently insufficient evidence to support the routine administration of immunostimulating nutrients (generally arginine, glutamine, omega-3 fatty acids, and/or nucleotides) in this group of patients^17^.

The inflammatory process creates a toxic environment for cells, as it renders the environment rich in oxygen free radicals that, associated with the overexpression of certain genes, lead to exponential damage to cellular DNA^28^ ^,^ ^29^. The inflammation that accompanies and grows along with tumors is also implicated in the sequence of events that lead the patient to weight loss, malnutrition, and cachexia, compromising treatment and, therefore, the prognosis of these patients^29^ ^-^ ^32^. Since McMillan’s publication, a few global services have started to adopt CRP and albumin measurements as mandatory in preoperative evaluation^12^. In Brazil, there is still no broad discussion on this topic, so these markers are not routinely requested by surgical services.

The main clinical application of our findings is that the use of GPC may guide the best choices of surgical teams, including opting for initially non-surgical therapies in patients with a high intensity of the inflammatory response, based on simple and widely available laboratory tests.

Importantly, there are different complication rates in different populations. Several factors interfere in this process, such as the protocols of each service to monitor, examine, assess risks, organize the health system to serve the population, among other variables^33^. This perhaps explains the different rates of postoperative complications between Western and Eastern studies^23^. This is exemplified by early detection in Asian countries where, in addition to diagnosing at a less advanced stage of the disease than in the West, do so at a much lower patient age than in the West at the time of diagnosis, which directly influences response to treatment^28^ ^,^ ^33^.

During the period of the SARS-COV-2 pandemic, the diagnosis of gastric cancer with the possibility of surgical treatment with curative intent was much lower than expected. There was also a significant reduction in the volume of elective surgeries, in addition to some patients not having CRP measured in the absence of an infectious condition and being excluded from the study at the beginning. These factors influenced the small sample size. Moreover, the surgical procedures were carried out in two hospitals, by different teams.

The measurement of CRP and albumin that are present in the GPC showed promise in predicting complications, which is worth discussing the need to include such parameters in the staging and mandatory preoperative surgical preparation in this disease and, possibly, in all malignant neoplasms. To corroborate this association between GPC and CD ≥ III-a, we suggest that more studies be carried out on the topic in the future, with a larger sample size, mainly prospective, with emphasis on randomized clinical trials.

## CONCLUSION

Regardless of the limiting factors mentioned above, this study showed that the Glasgow Prognostic Criteria predicted severe surgical complications in a controlled model for intraoperative and perioperative complications, represented by O-POSSUM and P-POSSUM. More in-depth studies are needed to implement strategies aimed at minimizing the inflammatory response in the preoperative period.
